# New Appliances and Things Medical

**Published:** 1894-05-12

**Authors:** 


### NEW APPLIANCES AND THINGS MEDICAL.

[All preparations, appliances, novelties, &c., of which a notice is desired, should be sent for the Editor, to care of The Manager, 428, Strand, London, W.C.]

### WINE OF COD LIVER OIL WITH PEPTONATE OF IRON AND OTHER PREPARATIONS (STEARNS').

(Thomas Christy and Co., 25, Lime Street, E.C.)

Samples of the above wine have been forwarded us by Messrs. Christy. The manufacturers of wine of cod liver oil disclaim the location of this preparation in the category of food stuffs. There was a time when everybody regarded cod liver oil as a food, and attached no further therapeutic value to its administration. According to authorities quoted by the manufacturers this is no longer the case; the virtue of cod liver oil in the treatment of phthisis and other wasting diseases depends not on its properties as an easily assimilated food, but on the presence of certain definite chemical principles, namely, volatile and fixed bases, as well as acid radicles, each of which has a definite pharmacodynamical action.

Their authorities for this statement are M. Gautier, M. Mourgues, and M. Bouilot, of Paris, who have separated from cod liver oil a large number of its component constituents, which constituents have been administered separately to animals, and the observed phenomena recorded.

Without doubting for an instant the accuracy of these observations, we do not feel inclined to accept their deductions, neither do we regard it a safe proceeding to draw such sweeping conclusions from such a limited supply of experimental data. Because the urine of guinea pigs has in a certain number of cases increased in quantity and become less turbid in appearance, and because the animals attack their food with greater avidity after the exhibition of morrhuic acid, these are no sufficient arguments for saying that the value of cod liver oil is dependent on the therapeutic effect of this constituent.

At the best of times the clinical results from the administration of cod liver oil are so slow and unconspicuous, that we should always feel inclined to regard with suspicion any strikingly beneficial effects reputed to be due to any of its component parts.

The wine of cod liver oil is stated to contain 25 per cent. of cod liver oil, as represented by the so-called "active constituents," with about 1 per cent. of peptonate of iron, and a considerable proportion of alcohol. It is not disagreeable as a medicine, and it is never followed by disagreeable after-effects such as eructation, nausea, &c., which often follow the ingestion of the simple oil.

We have tried it in one or two cases of pulmonary consumption, when the ordinary oil could not be tolerated. In each case the patient spoke of it enthusiastically, both as regards taste and stimulating effect. And the influence on nutrition as observed after a short course of the wine was decidedly favourable. Whether this was due to peptonate of iron, the alcohol, or the so-called active principles of the oil we hesitate to say.

### CASCARA AROMATICA (STEARNS).

This is a fluid extract of cascara, said to be made solely from two-year-old bark; the bitter principles, which give the ordinary preparations their disagreeable taste and griping after effect, have been removed, without in any way interfering with the laxative effect of the bark. For delicate children and sensitive women it is a great improvement on the old form.

### DIKES' PEPSIN (STEARNS).

This appears to be an excellent sample of the gastric ferment; it is composed of thin scales, very soluble in water, forming a clear solution, its strength is 1 in 3,000, and it has no disagreeable taste.

### THE "VERA" FEEDING BOTTLE.

(Edward Garthwaite Farish, 4, Elm Park Gardens.)

A specimen of the above bottle has been forwarded us for inspection. It is a feeding bottle of most novel features. If we may so describe it, it is constructed on the principle of a Norwegian stove. It fits into a case of stout leather lined with thick felt. The case is fitted with two straps, which, while keeping the bottle securely closed in its carapace, allow a ready inspection of the contents, or an exchange of bottle. The advantages of the contrivance are as follows: (1) The bottle cannot be broken by any ordinary degree of rough usage; (2) the contents are preserved at an equable temperature. We have, however, two suggestions to make which we hope may be of advantage in the further development of this embryo bottle. Firstly, there should be a fenestrum or window in the outer case to indicate when the contents of the bottle are becoming exhausted. Secondly, the form of bottle is a bad one. The long indiarubber tube should be dispensed with, and the teat should be in direct continuity with the bottle. Otherwise the invention is a clever and a useful one, and is bound to find many supporters.

### VIROL.

(Liquor Carnis Company, 28a, Farringdon Street, London, E.C.)

Virol is described as a highly concentrated complete food, consisting of proteids of beef and eggs, the fats of beef and eggs, the marrow of beef, the carbo-hydrate in the form of extract of malt, and the salts of beef and egg (including the lime salts of the shell). It is intended to supersede cod-liver oil. It has, indeed, many advantages over this oil. Firstly, it is palatable; secondly, it contains representatives of all those food stuffs which are included in a physiological diet; and thirdly, each of these representatives is offered in a form partly digested and easy of assimilation.

On theoretical grounds alone a physician would be justified in prescribing Virol as a food for the most delicate digestion, and even in cases of gastric ulcer. Where any form of fluid can be tolerated it might be safely administered, and clinical experience quite bears out this assumption.

Virol, indeed, is a most valuable addition to invalid dietaries; the danger in its application, however, is lest its use should be abused. Thus, it must be employed as an auxilliary to, and not as a substitute for, ordinary food. It is directed to be taken in teaspoonfuls; to serve as an independent and sufficient food something like three pounds would have to be taken during the twenty-four hours. And then the proteid factor in the food would be very deficient. The proportion of proteid, fats, and carbo-hydrate, as existing in Virol, according to our views, hardly justify the statement that they are "carefully adjusted to diet formulæ laid down by the most up-to-date physiologists." The fat is excessive according to this standard, and the proteid very deficient. If regarded merely as a substitute for cod-liver oil we cannot cavil at these proportions, but as a physiologically complete food we must protest against the above assertion. Virol is in appearance something like honey, it may be spread on bread or taken plain; it has an agreeable taste not altogether unlike that of toffee.

### INVALID BOVRIL.

(Essence of Beef—Brand Bovril. Bovril Company, 30, Farringdon Street, E.C.)

The Bovril Company have certainly taken a wise step in adapting their well-known fluid beef to the exacting requirements and capricious tastes of invalids. Invalid Bovril is made on the same principle as that employed in the manufacture of the ordinary kind, that is to say, not only are the extractives, but also the albuminous and nutritive constituents of beef are rendered in a soluble form, thus constituting a preparation which is at once a food and a stimulant. Invalid Bovril differs from the ordinary variety in the flavouring or seasoning. Although very palatable, it lacks the pepper and other condiments which in Bovril proper may irritate or inflame gastric mucous membrane or the delicate cells of the liver and portal system. As a nitrogenous and nutritious food for invalids it is probably without a rival. The Essence of Beef, which is prepared by the same company, is a most delicious beef jelly. When taken cold it is most happily adapted for cases of gastric ulcer, since it is nutritious and easily digested by the gastric secretions. This invalid speciality is stated to be prepared from Scotch beef. The juice is extracted by gentle heat, a process occupying about eight hours. After it has been clarified, a beautiful clear amber-coloured jelly is the result.

### ANTISEPTIC HANDKERCHIEFS, NEW PREPARATIONS, &c.

(Arthur and Co., 74, Newman Street, London, W.)

The antiseptic handkerchiefs which have been forwarded us by the above firm appeal to us as useful inventions. They are composed of thin, soft, Japanese paper, such as is sometimes used for serviettes, &c., and after use they should at once be put in the fire. They are especially intended for use in cases of phthisis, since the sputum of consumptives is often loaded with the tubercle bacilli, which, should it be allowed to dry on a handkerchief, may be disseminated through the air, and possibly inhaled by healthy persons, and finding lodgment in the lungs or elsewhere, form the foci of tubercular lesions. There is no question that consumptives are very careless in the manner in which they expectorate in the public streets or hospitals. They should, however, be taught the danger of this proceeding, and the possession of destructable handkerchiefs might be a constant reminder of the source of danger their sputum is to others. The sample we have inspected has not been treated in any way, but the manufacturers assure us that the specimens which they are now supplying are dipped in 1 in 2,000 and in 1 in 5,000 perchloride of mercury, and afterwards treated with eucalyptol and pinol. This should render them antiseptic as far as the surface of the sputum in contact with the handkerchief is concerned. But they should constantly be renewed and burnt at once after use. In cases of measles with coryza, and influenza with nasal catarrh, their use is probably more strongly indicated than in cases of consumption. The washable handkerchief is a most dangerous instrument for the dissemination of infectious disease. We cannot too strongly condemn its use in the sick-room. Destructable handkerchiefs, whether paper, cotton, or linen, are a wise and judicious prophylactic measure. The paper variety which we have above described costs less than does the washing of ordinary handkerchiefs. The samples of new preparations by the same firm include the following: (1) Ung. Hamamelidis co., in collapsible tubes, with introducer for hæmorrhoids; (2) massage unguent, composed of deodorised and softened oil of theobromine; (3) sedative snuff, for influenza and nasal catarrh; (4) extract. fuci. vesic. co.; (5) ol. morrhui etheris, a pure limpid oil; (6) liniment tannici co., strongly recommended for unhealthy perspiration of the feet; (7) mistura calcis chloridi cum ferro, a good form for administering iron, since it contains hardly any free acid. The bottles, corks, and water used in the preparation of the above drugs are all carefully sterilised; and a sterilised solution of morphia in small glass capsules in a convenient form for carrying is also supplied by the same firm. All the above drugs are first rate of their kind, and we do not hesitate to recommend them.

